# Intrinsic Functional Connectivity Alterations of the Primary Visual Cortex in Primary Angle-Closure Glaucoma Patients before and after Surgery: A Resting-State fMRI Study

**DOI:** 10.1371/journal.pone.0170598

**Published:** 2017-01-25

**Authors:** Shenghong Li, Ping Li, Honghan Gong, Fei Jiang, Dan Liu, Fengqin Cai, Chonggang Pei, Fuqing Zhou, Xianjun Zeng

**Affiliations:** 1 Department of Radiology, The First Affiliated Hospital, Nanchang University, Nanchang, Jiangxi Province, China; 2 Jiangxi Province Medical Imaging Research Institute, Nanchang, Jiangxi Province, China; 3 Department of Ophthalmology, The First Affiliated Hospital, Nanchang University, Nanchang, Jiangxi Province, China; Chinese Academy of Sciences, CHINA

## Abstract

**Purpose:**

To investigate the altered intrinsic functional connectivity (iFC) of the primary visual cortex (V1) in primary angle-closure glaucoma (PACG) patients before and after surgery using resting-state functional MRI.

**Materials and Methods:**

Twenty-five preoperative PACG (pre-PACG) patients and 25 well-matched healthy controls (HCs) were included in this study, and 9 PACG patients were assessed again at least 3 months after treatment (post-PACG). We generated the iFC maps of the seed regions in the centers of the left and right V1 and conducted group comparisons. Then, the relationships between the altered iFC coefficients and clinical variables were investigated in the pre-PACG patients.

**Results:**

Compared with the HCs, the pre-PACG patients showed decreased iFC between the left V1 and right V2 (covering the cuneus, calcarine and lingual gyrus) and increased iFC between the left V1 and left temporal-parietal region, left frontal opercula-insula-basal ganglia region, right insula-basal ganglia region, and right inferior parietal lobule (*P* < 0.01, corrected). Compared with the pre-PACG patients, the post-PACG patients showed increased iFC between the left V1 and bilateral V2, and between the left V1 and left or right postcentral gyrus; in addition, they showed decreased iFC between the left V1 and the dorsal-attention and frontoparietal-control networks. In the pre-PACG patients, visual activity (VA) was positively correlated with increased iFC between the left V1 and the left temporal-parietal region or the right inferior parietal lobule. Similar patterns of alterations were observed in the right V1-iFC in both the pre- and post-PACG patients.

**Conclusions:**

The primary findings have demonstrated a gradual decrease in visual information integration in the left V1-V2 pathway and VA-related functional compensation in the pre-PACG patients, generating further evidence of functional restoration in post-PACG patients.

## Introduction

Primary glaucoma, the second leading cause of blindness worldwide, will have affected 79.6 million people by 2020 [1]. Increased intraocular pressure (IOP) is considered a major risk factor for this disease in association with age. The progressive loss of retinal ganglion cells (RGCs) is thought to be a key factor for the pathogenesis of glaucoma [2–5]. However, advanced techniques, such as noninvasive neuroimaging, have demonstrated structural or functional alterations in the intracranial optic nerve and optic radiation [6], the lateral geniculate nucleus (LGN) [7], the visual cortex [8], and even outside the visual system, as well in patients with primary glaucoma [9].

Previous MRI studies have demonstrated that glaucoma patients exhibit not only neuronal degeneration in the geniculocalcarine tract and striate area [10] but also abnormal blood perfusion in the primary visual cortex (V1) [11], with increased functional activation during the initial disease stage and decreased functional activation during the later stage in these regions [12]. Recently, resting-state functional MRI (rs-fMRI) has been increasingly used to explore intrinsic functional activity in glaucoma patients and has provided critical information in the search for pathological mechanisms. Several recent studies have shown that glaucoma patients possess abnormalities such as decreased intrinsic functional connectivity (iFC), both within the visual network [13] and beyond the visual system [9], as well as alterations in amplitude of low-frequency fluctuations (ALFFs) [14] or regional homogeneity (ReHo) [15]. These results have been reported for patients with primary open-angle glaucoma (POAG), which is most prevalent in Western societies. In Asia and China, however, the most common type is primary angle-closure glaucoma (PACG) [1,16,17], which is probably the leading cause of glaucomatous blindness in both eyes [16]. The pattern of visual field loss tends to differ between PACG and POAG [18], for instance, the peripapillary atrophy in PACG has a different relationship with the structural and functional optic disk changes than that in POAG [19]. Compared with POAG, fewer retinal nerve fiber layer sectors have significant structure-function correlations in PACG [20], suggesting differences in the pathophysiology of optic nerve damage and even the whole visual pathway between PACG and POAG. In patients with PACG, our group has reported decreased functional centrality in the visual system and increased degree centrality (DC) in cognitive-emotional processing regions [21]. However, DC is a measure of the topology of the architecture of the brain functional connectome [22], but it does not reflect the temporal correlation between spatially remote neurophysiological events. iFC, which is amenable to simple and straightforward interpretation, allows for measurement of connectivity between brain regions that share functional properties [23].

In this study, we sought to obtain evidence of iFC alterations in V1. The aim was to investigate the potential functional plasticity of V1 in PACG patients with trabeculectomy and peripheral iridectomy, both before and after surgery. V1 is the core of the visual network. It receives visual information from the LGN directly, forms complex functional connections with the senior visual cortex (Brodmann area 18 (BA18), BA19), and may be susceptible to injury. Seed-based iFC analysis may provide a much more precise and detailed perspective on the specific connectivity of V1 in PACG patients. Thus, we hypothesized that V1-iFC analysis would reveal (1) disrupted connectivity between V1 and higher visual areas in preoperative PACG (pre-PACG) patients and (2) partial functional restoration of V1-associated pathways post-operatively, with improvement in clinical symptoms. Our findings may provide a new perspective for understanding the underlying pathological and compensatory mechanisms in V1 in PACG patients before and after surgery.

## Materials and Methods

### 1.1 Subjects

Forty right-handed pre-PACG patients were recruited from October 2013 to October 2015 from the Department of Ophthalmology of the First Affiliated Hospital, Nanchang University, China. The inclusion criteria for the pre-PACG patients were as follows: (1) narrow anterior chamber angles in both eyes confirmed clinically by gonioscopy and slit-lamp examination; (2) visual field defects associated with glaucoma, such as tubular vision and nasal hemianopia; and (3) an optic disk cup-to-disc ratio > 0.6, as determined by funduscopic examination. The exclusion criteria for the pre-PACG patients were as follows: (1) diagnosis with another type of glaucoma, such as POAG or secondary glaucoma; (2) diagnosis with another ocular disease or an organic disorder affecting the optic pathway; (3) a history of brain trauma; (4) a history of an underlying disease, such as hypertension or diabetes; (5) a history of surgical treatment for glaucoma; (6) an age of greater than 65 years; (7) incomplete data from MRI scan or clinical assessment; and (8) head movement of greater than 2-mm maximum displacement in any of the x, y, or z directions or of greater than 2° angular rotation in any axis during rs-fMRI exam. Ultimately, 25 pre-PACG patients (10 males and 15 females, 39 to 63 years of age) were included in this study (6 patients were older than 65 years; 4 had incomplete clinical data; and 5 had head movement > 2 mm or > 2° during rs-fMRI exam).

We recruited and selected 25 right-handed, age- and gender-matched healthy subjects as controls (HCs; 10 males and 15 females, 39 to 65 years of age). The exclusion criteria for the HCs were as follows: (1) diagnosis with an ocular disorder or other systemic disease; (2) severe nearsightedness or farsightedness; (3) contraindications for MRI, such as metal implants or claustrophobia; (4) an age of greater than 65 years; and (5) head movement of greater than 2-mm maximum displacement in any of the x, y, or z directions or of greater than 2° angular rotation in any axis.

In this study, 25 PACG patients underwent trabeculectomy and peripheral iridectomy, but only 10 patients (post-PACG) were followed up for at least 3 months post-treatment due to poor patient compliance. One patient with head movement > 2 mm during MRI exam was excluded.

This study complied with the Declaration of Helsinki, and the Human Research Ethics Committee of the First Affiliated Hospital of Nanchang University approved the study protocol. Written informed consent was obtained from each participant prior to the study. Tables 1 and 2 provide demographic information for the pre- and post-PACG patients and HCs.

**Table 1:** General clinical information for preoperative primary angle-closure glaucoma (pre-PACG) patients and healthy controls (HCs).

Condition	Pre-PACG	HCs	*P* value
Mean age (years) (range)	52 (39-63)	52 (39-65)	0.86
Gender (male/female)	10/15	10/15	> 0.99
Handedness (right/left)	25/0	25/0	> 0.99
Mean disease duration (days) (range)	627 (2-3650)	-	n/a
IOP (mmHg)	31.12±11.42	-	n/a
RNFLT (μm)	85.24±19.54	-	n/a
A-C/D	0.65±0.17	-	n/a
V-C/D	0.63±0.21	-	n/a
Mean VA (range)	0.61 (0.04-1.25)	-	n/a
Head motion	0.048±0.014	0.039±0.013	0.582

Notes and Abbreviations: - = no data; IOP, intraocular pressure; RNFLT, retinal nerve fiber layer thickness; A-C/D, average cup-to-disc ratio; V-C/D, vertical cup-to-disc ratio; VA, visual acuity; n/a = not applicable; IOP, RNFLT, A-C/D, V-C/D, and VA are presented as mean binocular values. The same abbreviations are used for all figures and tables.

**Table 2:** General clinical information for postoperative primary angle-closure glaucoma (post-PACG) and corresponding preoperative-PACG (pre-PACG) patients.

Condition	Pre-PACG	Post-PACG	*P* value
Mean age (years) (range)	53 (43-63)	53 (43-63)	> 0.99
Gender (male/female)	3/6	3/6	> 0.99
Handedness (right/left)	9/0	9/0	> 0.99
IOP (mmHg)	32.56±7.02	17.33±1.80	0.03
RNFLT (μm)	88.22±18.99	85.94±18.27	0.79
A-C/D	0.67±0.14	0.68±0.13	0.94
V-C/D	0.64±0.17	0.65±0.17	0.96
Mean VA (range)	0.56 (0.04-1.10)	0.60 (0.08-1.20)	0.81
Head motion	0.051±0.011	0.036±0.015	0.213

Note: IOP, RNFLT, A-C/D, V-C/D, and VA are presented as mean binocular values.

### 1.2 Data acquisition

The rs-fMRI data were acquired using a 3-T MR scanner (Siemens, Erlangen, Germany) with an 8-channel phased-array head coil at the Department of Radiology of the First Affiliated Hospital, Nanchang University, China. All subjects were directed to sit for 10 minutes prior to resting-state scanning. Next, they were instructed to keep their eyes closed but to not fall asleep. The subjects were further instructed to not engage in any specific thinking activities during data acquisition. Head movements and noise were suppressed using a suitable sponge mat and earplugs, respectively. rs-fMRI data acquisition lasted for 8 minutes, and 240 resting-state volumes were acquired using the following parameters: repetition time (TR) = 2000 ms; echo time (TE) = 40 ms; flip angle = 90°; field of view (FOV) = 240 mm×240 mm; matrix = 64×64; and slice thickness = 4 mm with 1 mm gap. Each brain volume included 30 axial slices. High-resolution T1-weighted images for each subject were acquired with a 3D MRI sequence, and the parameters were as follows: TR = 1900 ms; TE = 2.26 ms; flip angle = 9°; FOV = 240 mm×240 mm; matrix = 256×256; number of sagittal slices = 176; and slice thickness = 1 mm.

### 1.3 Data preprocessing

We utilized DPARSFA advanced edition (DPARSFA 4.0, http://rfmri.org/DPARSF)) which was based on statistical parametric mapping (SPM, http://www.fil.ion.ucl.ac.uk/spm) and the rs-fMRI Data Analysis Toolkit (REST, http://www.restfmri.net) for the rs-fMRI data analysis [24]. The main preprocessing procedure was as follows: (1) transformation of DICOM files into NIFTI images; (2) discarding of the first 10 volumes to allow for magnetization equilibration and adaptation of the subjects to the surroundings; (3) slice timing; (4) head motion correction; (5) co-registration of functional images to high-resolution T1-weighted structural images, segmentation of structural data, spatial normalization to standard Montreal Neurological Institute (MNI) space, and resampling to 3×3×3 mm isotropic voxels; (6) smoothing with a 6 mm full-width-half-maximum Gaussian kernel; (7) removal of the linear trend of the time series; (8) filtering (0.01–0.1 Hz) to reduce the effects of low-frequency drifts and high-frequency noise; and (9) regressing out head motion as well as white matter, cerebrospinal fluid and global signals as nuisance variables. Some studies have demonstrated that higher-order models are beneficial for the removal of head motion effects; therefore, the Friston 24-Parameter Model was used to regress out these effects in this study. We only regressed out the global signal to obtain the iFC patterns because it is unclear whether global signal regression has complex effects on functional connectivity [25]. The head motion data are shown in Tables 1 and 2 and are based on computation of voxel-specific frame-wise displacement (FDvox) with the DPARSFA toolbox according to the criteria of Van Dijk et al. [26].

### 1.4 Seed-based iFC analysis

Regions of interest (ROIs) were selected according to the literature [13]. The center of V1 was chosen as the seed point, and the MNI coordinates of bilateral V1 were (left: -8,-76,10) and (right: 7,-76,10). The diameter of the sphere ROI was 10 mm (approximately 27 cubic voxels), and Pearson correlation coefficients were calculated between the mean time course of the ROI and the time courses for all other brain voxels. Fisher’s z transform analysis was applied to the Pearson correlation coefficients to obtain an approximate normal distribution to enable the subsequent statistical analysis.

### 1.5 Clinical assessment

All patients underwent detailed ophthalmological examination. The state of the anterior chamber angle was determined by gonioscopy and the slit-lamp examination. IOP was measured using a tonometer, while retinal nerve fiber layer thickness (RNFLT), the average cup-to-disc ratio (A-C/D) and the vertical cup-to-disc ratio (V-C/D) were evaluated by optical coherence tomography (Cirrus HD-OCT). In addition, the disease course and visual acuity (VA) were recorded.

### 1.6 Statistical analysis

Statistical analysis of the general clinical data was performed using SPSS software version 19.0 (IBM, Armonk, NY, USA), and Student’s t-test was used to examine differences in age, gender, handedness, and ophthalmic findings between the pre-PACG patients and HCs. The paired-sample t-test was also used for group comparisons between the post- and pre-PACG patients (*P* < 0.05). Further, the Chi-square test was used for comparisons of categorical data (gender and handedness).

The statistical module of the dpabi (http://rfmri.org/dpabi) was used for rs-fMRI data analysis. The random-effect one-sample t-test was used to determine the spatial distribution pattern of iFC of V1 in the HCs, and pre- and post-PACG groups (two-tailed false discovery rate (FDR)-corrected *P* < 0.001). The iFC maps were compared using a general linear model (GLM) and one-way analysis of covariance (ANCOVA), with age and gender used as covariates. The post hoc, two-sample t-test was used to examine differences in iFC between the pre-PACG patients and HCs (one-tailed Gaussian random field (GRF)-corrected at voxel level *P* < 0.01 and cluster level *P* < 0.05). In addition, the paired-sample t-test was used to examine differences in iFC between the post- and pre-PACG groups (one-tailed GRF-corrected voxel-level *P* < 0.01 and cluster-level *P* < 0.05).

Clarification of the different statistical correction methods used in rs-fMRI data analysis: (1) in the one-sample t-test, FDR correction was used to reduce type I errors; (2) in the post hoc two-sample t-test, GRF correction was used for group comparisons, for which correction has been used extensively in the fMRI literature. Controlling the FDR could provide a more severe and rigorous analysis of the FDR of voxels than GRF correction for family-wise errors, but for group comparison, it should be controlling the FDR of features. In the brain, neuronal activity propagates through intrinsic and lateral connections, and the activity signal recorded by the BOLD effect was used as smoothing for between-subject (second-level) analyses with Gaussian filters, rendering a continuous signal. The GRF correction has a clear advantage because it uses a smoothness estimator prior to the analysis to reduce the number of independent voxels (or, rather, resels, i.e. resolution elements) [27–29]. If the FDR correction was instead performed for group comparisons, there would be few voxels.

Finally, *Pearson* correlation (multiple linear regression in SPSS) analysis was performed for the PACG patients to assess the correlations between the clinical variables (disease duration, IOP, RNFLT, A-C/D, V-C/D, and VA) and the average iFC coefficient measured in each region, which showed differences between the groups (with age and gender as covariates, multiple comparisons performed using a Bonferroni-corrected *P* < 0.0083).

## Results

### 2.1 General clinical information

As shown in Table 1, no significant differences were detected in age, gender, or handedness between the pre-PACG patients and HCs (*P >* 0.05). In addition, as shown in Table 2, no significant differences were observed in the ophthalmic findings, except for IOP, between the pre- and post-PACG patients.

### 2.2 Spatial distribution and alteration of iFC of V1 in pre-PACG patients

**Fig 1:**
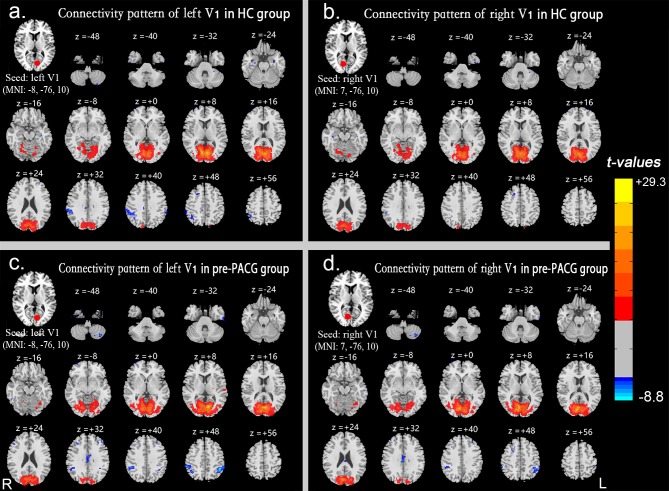
iFC spatial distribution maps of V1. iFC spatial distribution maps of the left (**a**) and right (**b**) V1 in the HCs and the left (**c**) and right (**d**) V1 in the pre-PACG patients based on a threshold of *P* < 0.001 with false discovery rate correction. Spatial distribution was visualized using DPABI slice viewer (http://rfmri.org/dpabi). Abbreviations: iFC, intrinsic functional connectivity; V1, primary visual cortex; HCs, healthy controls; pre-PACG, preoperative primary angle-closure glaucoma. The same abbreviations are used for all figures and tables.

The iFC spatial distribution maps of the left or right V1 in the HCs (n = 25) and pre-PACG patients (n = 25) were generated by seed-based functional connectivity analysis (Fig 1A–1D). The statistical significance of each result was based on a threshold of *P* < 0.001 with the FDR corrected.

**Fig 2:**
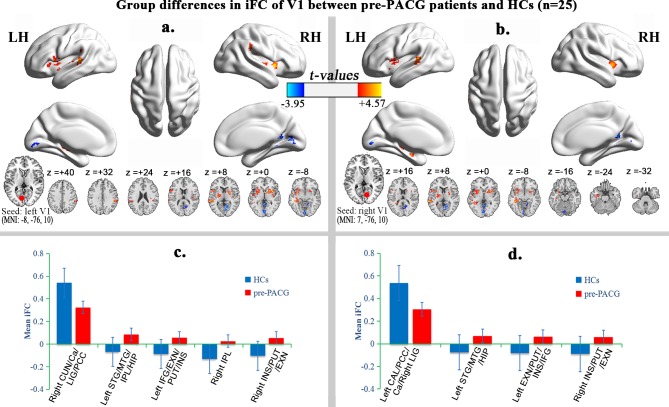
Alteration of iFC of V1 in pre-PACG patients. Regions showing significant differences in iFC of the left (**a**) and right (**b**) V1 between the pre-PACG patients and HCs (one-tailed voxel-level *P* < 0.01 and cluster-level *P* < 0.05 with GRF correction). The hot (cool) color represents increased (decreased) iFC in the pre-PACG patients compared with the HCs. Spatial distribution was visualized using surface brain imaging in BrainNet Viewer (www.nitrc.org/projects/bnv/) and DPABI slice viewer (http://rfmri.org/dpabi). Bar plot of the iFC of the left V1 (**c**) and the right V1 (**d**) for the significant clusters in the pre-PACG patients vs. HCs.

Compared with the HCs, the pre-PACG patients (n = 25) exhibited significantly decreased iFC between the right cuneus/calcarine/lingual gyrus/posterior cingulate cortex (CUN/Ca/LIG/PCC) and the left V1. In addition, significantly increased iFC was observed between the left superior temporal gyrus/middle temporal gyrus/inferior parietal lobule/hippocampus (STG/MTG/IPL/HIP), left inferior frontal gyrus/extra-nuclear/putamen/insula (IFG/EXN/PUT/INS), right IPL, and right INS/PUT/EXN, and the left V1 (Table 3 and Fig 2A and 2C).

**Table 3:** Group differences in iFC of V1 between pre-PACG patients and HCs (one-tailed voxel-level *P* < 0.01 and cluster-level *P* < 0.05 with GRF correction).

Brain regions	Voxels	MNI coordinates			BA	*T* value
		X	Y	Z		
**Seed: left V1**						
Right CUN/Ca/LIG/PCC	252	9	-72	9	18,30	-3.95
Left STG/MTG/IPL/HIP	243	-39	-21	-12	22	4.57
Left IFG/EXN/PUT/INS	287	-45	12	12	44	3.91
Right IPL	141	54	-33	33	40	3.81
Right INS/PUT/EXN	218	36	9	-3	-	4.52
**Seed: right V1**						
Left CAL/PCC/Ca/Right LIG	227	-3	-57	-3	18	-3.68
Left STG/MTG/HIP	316	-42	-36	-6	22	4.49
Left EXN/PUT/INS/IFG	318	-21	3	18	-	4.14
Right INS/PUT/EXN	178	39	9	3	-	4.17

Abbreviations: MNI, Montreal Neurological Institute; BA, Brodmann area; CUN, cuneus; Ca, calcarine; LIG, lingual gyrus; PCC, posterior cingulate cortex; STG, superior temporal gyrus; MTG, middle temporal gyrus; IPL, inferior parietal lobule; HIP, hippocampus; IFG, inferior frontal gyrus; EXN, extra-nuclear; PUT, putamen; INS, insula; CAL, cerebellum anterior lobe. The same abbreviations are used for all figures and tables.

Compared with the HCs, the pre-PACG patients (n = 25) exhibited significantly decreased iFC between the left cerebellum anterior lobe (CAL)/PCC/Ca/right LIG and the right V1. Moreover, significantly increased iFC was detected between the left STG/MTG/HIP, the left EXN/PUT/INS/IFG, and right INS/PUT/EXN, and the right V1 (Table 3 and Fig 2B and 2D).

### 2.3 Alteration of iFC of V1 between pre-PACG and post-PACG patients

**Fig 3:**
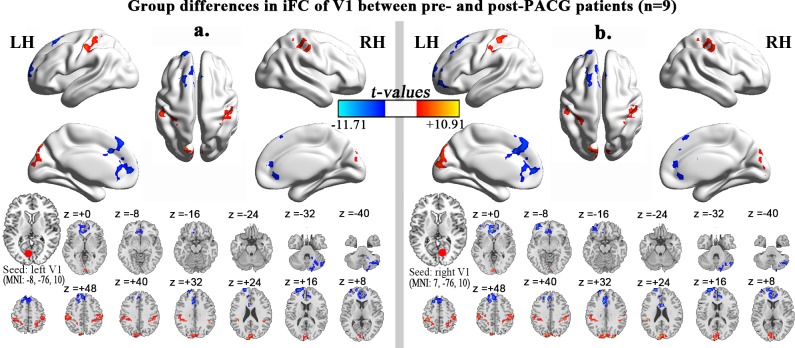
Alteration of iFC of V1 between the pre-PACG and post-PACG patients. Regions showing significant differences in iFC of the left V1 (**a**) and right V1 (**b**) between the post- and pre-PACG patients (one-tailed voxel-level *P* < 0.01 and cluster-level *P* < 0.05 with GRF correction). The hot (cool) color represents increased (decreased) iFC in the post-PACG patients compared with the pre-PACG patients. Spatial distribution was visualized using surface brain imaging in the BrainNet Viewer (www.nitrc.org/projects/bnv/) and DPABI slice viewer (http://rfmri.org/dpabi).

Compared with the pre-PACG patients (n = 9), the post-PACG patients (n = 9) exhibited significantly decreased iFC between the left superior frontal gyrus/anterior cingulate cortex/middle frontal gyrus (SFG/ACC/MFG), right cerebellum posterior lobe (CPL) and the left V1. In addition, significantly increased iFC was observed between the left precuneus (PCU)/CUN/right superior occipital gyrus (SOG), left and right postcentral gyrus (POCG)/IPL and the left V1 (Table 4, Fig 3A).

**Table 4:** Group differences in iFC of V1 between post- and pre-PACG patients (one-tailed voxel-level *P* < 0.01 and cluster-level *P* < 0.05 with GRF correction).

Brain regions	Voxels	MNI coordinates			BA	*T* value
		X	Y	Z		
**Seed: left V1**						
Left SFG/ACC/MFG	602	-18	63	21	10,32	-11.70
Right CPL	306	51	-69	-42	-	-7.03
Left PCU/CUN/Right SOG	302	-18	-81	42	18,19,7	10.91
Left POCG/IPL	368	-51	-24	48	2,40	6.65
Right POCG/IPL	391	57	-21	48	2,40	6.05
**Seed: right V1**						
Left ACC/MFG/SFG	1298	-12	36	-3	10,32	-9.58
Right CPL	198	51	-69	-42	-	-9.54
Left CUN/Right SOG/Ca	459	-9	-87	45	18,19	8.66
Left POCG/IPL	337	-39	-36	57	2,40	6.44
Right POCG/IPL	388	57	-21	48	2,40	6.47

Abbreviations: MNI, Montreal Neurological Institute; BA, Brodmann area; SFG, superior frontal gyrus; ACC, anterior cingulate cortex; MFG, middle frontal gyrus; CPL, cerebellum posterior lobe; PCU, precuneus; SOG, superior occipital gyrus; POCG, postcentral gyrus. The same abbreviations are used for all figures and tables.

Compared with the pre-PACG patients (n = 9), the post-PACG patients (n = 9) showed significantly decreased iFC between the left ACC/MFG/SFG, right CPL and the right V1. In addition, significantly increased iFC was detected between the left CUN/right SOG/Ca, left and right POCG/IPL and the right V1 (Table 4, Fig 3B). Further, group differences between the post-PACG patients and HCs were observed in the regions with altered V1-iFC between the pre- and post-PACG patients (S1 Fig shows the results of an ANOVA comparing the HCs (n = 9), pre-PACG patients (n = 9), and post-PACG patients (n = 9); and S2 Fig shows the results of the two-sample t-test analysis between the HCs and post-PACG patients).

### 2.4 Relationships between clinical indices and iFC of V1 in pre-PACG patients

**Fig 4:**
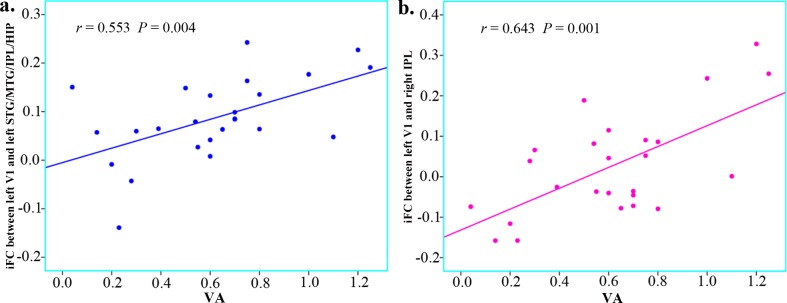
Relationships between clinical indices and iFC of V1 in pre-PACG patients. The increased iFC coefficients between the left STG/MTG/IPL/HIP and left V1 (**a**, *r* = 0.553, *P* = 0.004) and between the right IPL and left V1 (**b**, *r* = 0.643, *P* = 0.001) were positively correlated with VA.

In the pre-PACG group, correlation analysis between the clinical variables (disease duration, IOP, RNFLT, A-C/D, V-C/D, and VA) and iFC coefficients (between the regions with group differences in the left or right V1) showed that the increased iFC coefficients between the left STG/MTG/IPL/HIP and left V1 (*P* = 0.004), and between the right IPL and left V1 (*P* = 0.001) were positively correlated with VA (Fig 4). By contrast, no significant correlations were observed between the clinical indices and the other iFC coefficients for the pre-PACG patients (S1 and S2 Tables). In addition, no significant correlation was detected between the IOP and altered V1-iFC coefficients in the post-PACG patients (S3 Table).

## Discussion

The main finding of this study is that the pre-PACG patients exhibited decreased visual information integration in the left V1-V2 pathway and functionally relevant compensation in multimodal processing and visual-cognition regions. The significantly decreased iFC of the left V1 was distributed among the V2 (BA18) regions, mainly the right CUN/Ca/LIG, in the pre-PACG patients. However, the increased left V1-iFC was mainly distributed in the left temporal-parietal region (STG/MTG/IPL/HIP; in multimodal processing regions), left frontal opercula-insula-basal ganglia region (IFG/EXN/PUT/INS; in multimodal processing regions), right IPL, and right insula-basal ganglia region (INS/PUT/EXN). At three months after trabeculectomy and peripheral iridectomy, the post-PACG patients showed increased left V1-iFC, mainly distributed in the left PCU/CUN/right SOG (BA18, BA19, and BA7) and bilateral POCG, but decreased left V1-iFC in the prefrontal cortex and right CPL. Collectively, these changes are indicative of improved left V1-V2 integration. The pattern of alterations in right V1-iFC was similar to that of left V1-iFC in the pre- and post-PACG patients.

### 3.1 Evidence of functional plasticity in the left V1-V2 pathway in both pre- and post-PACG patients

The results of this study showed that the pre-PACG patients exhibited significantly decreased iFC of the left V1-V2 pathway, but these decreased iFC values were significantly improved in the post-PACG patients (see S3 Fig).

In the PACG patients, the blocked outflow of aqueous humor and increased IOP led to the degeneration or apoptosis of RGCs and cross-synaptic degeneration in the visual pathway [8,30], resulting in reduced visual information input and/or dysfunction in the central visual cortex. Recently, many rs-fMRI studies have shown reduced spontaneous brain activity in the central visual cortex in glaucoma patients, including decreased ALFF values in the bilateral CUN, Ca and left LIG [14], decreased ReHo values in the bilateral Ca and right LIG [15], and decreased DC values in V1 and V2 [21]. This evidence of reduced intrinsic activity implies dysfunction of the V1-V2 pathway. In the present study, decreased iFC was demonstrated in the left V1-V2 pathway by seed-based connectivity analysis. Significantly decreased iFC was observed between the left V1 and right V2 (mainly covering the CUN, Ca and LIG) in the pre-PACG patients (treatment-naïve), indicating reduced visual information integration. In a previous study, Dai et al. [13] have shown decreased iFC between V1 and the left fusiform gyrus, left middle occipital gyrus, and right SOG in POAG patients. These brain regions are located in BA19, which is also part of V2. The findings of our study are similar to those of Dai et al.’s study, but they are not identical for POAG patients because patients with different types of glaucoma were involved in the two studies.

Interestingly, by three months after treatment, the iFC between the left V1 and V2 was significantly increased, indicating improved intrinsic activity of the left V1-V2 pathway post-treatment by trabeculectomy and peripheral iridectomy. These results are consistent with those of previous study of DC [21]. In our study, although the visual field loss was irreversible, almost all of the PACG patients (9/9 post-PACG) exhibited slightly ameliorated clinical symptoms or visual function (significantly decreased IOP and slightly improved VA). It has been reported that at lower IOP, patients show better retention of visual function [31]. Our findings demonstrated significantly decreased IOP after surgical treatment, resulting in a reduction in the degeneration or apoptosis of RGCs, which led to a decrease in cross-synaptic degeneration in the visual cortex. This process finally culminated in functional plasticity of the visual cortex and V1-V2 pathway, and it plays an important role in the promotion/recovery of the visual function of PACG patients after surgery.

### 3.2 Compensatory increase in functional connectivity in pre-PACG patients

In this study, we observed increased iFC between the left V1 and the left temporal-parietal region (STG/MTG/IPL/HIP) and left frontal opercula-insula-basal ganglia region (IFG/EXN/PUT/INS), as well as between the right IPL and the right insula-basal ganglia region (INS/PUT/EXN), indicating functional compensation in the pre-PACG patients.

Our findings showed that the IPL and left HIP, part of the default-mode network (DMN), are functionally connected to the visual cortex. Functional connectivity analysis of human fMRI data revealed that visual cortical areas that selectively process relevant information are functionally connected to the frontal-parietal network, while those processing irrelevant information are simultaneously coupled with the DMN [32]. More importantly, the DMN is an intrinsically visually oriented system [33]. In the PACG patients, the decreased visual monitoring ability was inevitable because of the visual field defects and abnormal VA. Increased iFC between the left V1 and DMN (IPL and left HIP), particularly iFC between the left V1 and right IPL, was positively correlated with VA, suggesting functional compensation of the DMN to improve VA.

The STG is related to auditory information processing, the MTG is associated with auditory and language processing, the IFG is the correlational language cortex, and the INS is part of the frontal attention network [34]. Altered iFC values have been reported in POAG patients between the visual cortex and the right STG, right INS, and EXN [13]. In the PACG patients, the increased iFC values between the left V1 and left STG/MTG, left IFG, and right INS indicated increased visual information input from the visual cortex to the auditory, language and frontal attention networks, which could be explained by functional compensation due to a loss of inhibitory input from V1 or compensatory recruitment. The increased iFC between the left V1 and the left STG/MTG/IPL/HIP association with enhanced VA indicates that these compensatory values might contribute to the improvement of VA.

### 3.3 Functional restoration in post-PACG patients

Significantly increased iFC between the left V1 and POCG (primary somatosensory cortex, belonging to the somato-motor network [34]) was observed in the post-PACG patients. The somato-motor network had been reported to be associated with V1 spontaneous activity [35], and in a previous study, decreased iFC between V1 and POCG has been observed in POAG patients [13]. In glaucoma patients, a reduction in the degeneration or apoptosis of RGCs can occur in post-surgery patients, along with decreased IOP and improved visual function. Under these conditions, the visual cortex may receive more visual information to visually guide somatosensory information processing, resulting in increased functional connectivity between V1 and the somatosensory cortex.

Decreased iFC of the left V1 was also detected in the post-PACG patients. These brain regions mainly included the left SFG/MFG and right CPL. The MFG and right CPL belong to the dorsal attention network, and the left SFG is part of the frontoparietal control network [34]. Decreased iFC between V1 and the attention or control network reflects the decreased transmission of visual-related information originating in V1 or high-efficiency information exchange in the post-PACG patients, with improved IOP or visual sense. An alternative interpretation is decreased functional compensation in the frontal lobe in the post-PACG patients, with visual functional improvement or plasticity of the visual cortex.

### 3.4 Similar alteration in right V1-iFC in pre- and post-PACG patients

In this study, the patterns of iFC alterations in right V1 were similar to those in the left V1 in the pre- and post-PACG patients relative to the controls. Compared with the pattern of iFC alterations in the left V1, more regions with decreased iFC in the right V1 were detected in the left CAL in the pre-PACG patients. The CAL is the portion of the cerebellum responsible for mediating unconscious proprioception, and it may play a critical role in the execution and proper timing of learned responses [36]. Decreased FC between the V1 and the cerebellum has been reported in amblyopia [37], indicating that visual impairment and reduced visual information input might impact the CAL- mediated executive control function. In this study, we observed decreased iFC of the right V1-left CAL in the pre-PACG patients, verifying the previous finding of decreased iFC in amblyopia patients and indicating a visual-related executive control dysfunction in pre-PACG patients.

## Limitations

This study has several limitations. First, neuropsychological assessment should be conducted because depression, mental disorders and other mental symptoms have been observed in a large number of PACG patients. Second, the number of post-PACG patients was relatively low because of poor patient compliance; therefore, more post-PACG patients should be followed up and the iFC alterations at different stages after surgery should be further studied to explore the neuroplastic trajectories of surgery. Third, the disease durations of the pre-PACG patients varied greatly. Finally, in many pre-PACG patients, medication was used to lower IOP, which might have affected the iFC of V1 to some extent.

## Conclusions

This study characterized the iFC of V1 in patients with PACG. The primary findings indicated decreased visual information integration in the left V1-V2 pathway and VA-related functional compensation in multimodal processing and visual-cognition regions in the pre-PACG patients, generating further evidence of functional restoration in post-PACG patients. These findings provide insight for increasing the understanding of the underlying pathological and compensatory mechanisms in the central nervous system in PACG patients before and after surgery.

## Supporting Information

S1 Dataset iFC maps of the left V1 for the HCs and pre-PACG patients (n = 25). (ZIP)

S2 Dataset iFC maps of the right V1 for the HCs and pre-PACG patients (n = 25). (ZIP)

S3 Dataset iFC maps of the left V1 for the HCs and pre- and post-PACG patients (n = 9). (ZIP)

S4 Dataset iFC maps of the right V1 for the HCs and pre- and post-PACG patients (n = 9). (ZIP)

S1 Fig **ANOVA of the iFC of the left V1 (a) and right V1 (b) in the HCs, and pre- and post-PACG patients (n = 9).** The hot color indicates regions showing significant differences in iFC of the left (**a**) and right (**b**) V1 among the HCs, pre- and post-PACG patients (one-tailed voxel-level *P* < 0.01 and cluster-level *P* < 0.05 with GRF correction). These brain regions mainly included the right CPL, left SFG/MFG/ACC, left or right CUN/LIG/Ca/SOG, left PCU/PCC, and left or right POCG/IPL. Spatial distribution was visualized using DPABI slice viewer (http://rfmri.org/dpabi).
(TIF)

S2 Fig **Alterations of iFC of the left V1 (a) and right V1 (b) between the HCs and post-PACG patients (n = 9).** The hot (cool) color indicates increased (decreased) iFC of the left V1 (**a**) and right V1 (**b**) in the post-PACG patients compared with the HCs. Compared with the HCs, the post-PACG patients exhibited significantly increased iFC between the left or right CUN/right SOG/PCU/superior parietal lobule/left LIG/Ca, right INS/POCG/precentral gyrus (PRCG)/IPL, left POCG/PRCG/IPL/STG and the left V1; In addition, significantly decreased iFC was observed between the left or right CPL, left or right SFG/medial frontal gyrus/MFG/ACC, left or right PCU/PCC and the left V1. The iFC alterations of the right V1 was similar to those of the left V1. Spatial distribution was visualized using DPABI slice viewer (http://rfmri.org/dpabi).
(TIF)

S3 Fig Bar plot of iFC of the left V1 (a) and right V1 (b) for the significant clusters in the post-PACG patients vs. pre-PACG patients vs. HCs (n = 9).
(TIF)

S1 Table Correlation analysis between the clinical indices and altered left V1-iFC coefficients in the pre-PACG patients. (DOC)

S2 Table Correlation analysis between the clinical indices and altered right V1-iFC coefficients in the pre-PACG patients. (DOC)

S3 Table Correlation analysis between the IOP and altered V1-iFC coefficients in the post-PACG patients. (DOC)

## References

[1] Quigley HA, Broman AT (2006) The number of people with glaucoma worldwide in 2010 and 2020. Br J Ophthalmol 90: 262–267. 10.1136/bjo.2005.081224 16488940 PMC1856963

[2] Kwon YH, Fingert JH, Kuehn MH, Alward WL (2009) Primary open-angle Glaucoma. N Engl J Med 360: 1113–1124. 10.1056/NEJMra0804630 19279343 PMC3700399

[3] Morgan JE, Uchida H, Caprioli J (2000) Retinal ganglion cell death in experimental glaucoma. Br J Ophthalmol 84: 303–310. 10.1136/bjo.84.3.303 10684843 PMC1723413

[4] Holcombe DJ, Lengefeld N, Gole GA, Barnett NL (2008) Selective inner retinal dysfunction precedes ganglion cellloss in a mouse glaucoma model. Br J Ophthalmol 92: 683–688. 10.1136/bjo.2007.133223 18296504

[5] Desatnik H, Quigley HA, Glovinsky Y (1996) Study of central retinal ganglion cell loss in experimental glaucoma in monkey eyes. J Glaucoma 5: 46–53. 8795733

[6] Zhang QJ, Wang D, Bai ZL, Ren BC, Li XH (2015) Diffusion tensor imaging of optic nerve and optic radiation in primary chronic angle-closure glaucoma using 3T magnetic resonance imaging. Int J Ophthalmol 8: 975–979. 10.3980/j.issn.2222-3959.2015.05.22 26558212 PMC4631009

[7] Gupta N, Greenberg G, de Tilly LN, Gray B, Polemidiotis M, Yücel YH (2009) Atrophy of the lateral geniculate nucleus in human glaucoma detected by magnetic resonance imaging. Br J Ophthalmol 93: 56–60. 10.1136/bjo.2008.138172 18697810 PMC2605243

[8] Gupta N, Ang L, de Tilly LN, Bidaisee L, Yücel YH (2006) Human glaucoma and neural degeneration in intracranial optic nerve, lateral geniculate nucleus, and visual cortex. Br J Ophthalmol 90: 674–678. 10.1136/bjo.2005.086769 16464969 PMC1860237

[9] Frezzotti P, Giorgio A, Motolese I, De Leucio A, Iester M, Motolese E, et al (2014) Structural and Functional Brain Changes beyond Visual System in Patients with Advanced Glaucoma. PLoS One 9: e105931 10.1371/journal.pone.0105931 25162716 PMC4146554

[10] Zhang Y, Chen X, Wen G, Wu G, Zhang X (2013) Proton Magnetic Resonance Spectroscopy ((1)H-MRS) Reveals Geniculocalcarine and Striate Area Degeneration in Primary Glaucoma. PLoS One 8: e73197 10.1371/journal.pone.0073197 24009739 PMC3756940

[11] Duncan RO, Sample PA, Bowd C, Weinreb RN, Zangwill LM (2012) Arterial Spin Labeling fMRI Measurements of Decreased Blood Flow in Primary Visual Cortex Correlates with Decreased Visual Function in Human Glaucoma. Vision Res 60: 51–60. 10.1016/j.visres.2012.03.012 22465941 PMC3340501

[12] Gerente VM, Schor RR, Chaim KT, Felix Mde M, Ventura DF, Teixeira SH, et al (2015) Evaluation of Glaucomatous Damage via Functional Magnetic Resonance Imaging, and Correlations Thereof with Anatomical and Psychophysical Ocular Findings. PLoS One 10: e0126362 10.1371/journal.pone.0126362 25969982 PMC4430279

[13] Dai H, Morelli JN, Ai F, Yin D, Hu C, Xu D, et al (2013) Resting-state functional MRI: functional connectivity analysis of the visual cortex in primary open-angle glaucoma patients. Hum Brain Mapp 34: 2455–2463. 10.1002/hbm.22079 22461380 PMC6870365

[14] Li T, Liu Z, Li J, Liu Z, Tang Z, Xie X, et al (2015) Altered amplitude of low-frequency fluctuation in primary open-angle glaucoma: a resting-state FMRI study. Invest Ophthalmol Vis Sci 56: 322–329. 10.1167/iovs.14-14974 25525176

[15] Song Y, Mu K, Wang J, Lin F, Chen Z, Yan X, et al (2014) Altered spontaneous brain activity in primary open angle glaucoma: a resting-state functional magnetic resonance imaging study. PLoS One 9: e89493 10.1371/journal.pone.0089493 24586822 PMC3933564

[16] Foster PJ, Johnson GJ (2001) Glaucoma in China: how big is the problem. Br J Ophthalmol 85: 1277–1282. 10.1136/bjo.85.11.1277 11673287 PMC1723754

[17] Cheng JW, Zong Y, Zeng YY, Wei RL (2014) The Prevalence of Primary Angle Closure Glaucoma in Adult Asians: A Systematic Review and Meta-Analysis. PLoS One 9: e103222 10.1371/journal.pone.0103222 25057993 PMC4110010

[18] Gazzard G, Foster PJ, Viswanathan AC, Devereux JG, Oen FT, Chew PT, et al (2002) The severity and spatial distribution of visual field defects in primary glaucoma: a comparison of primary open-angle glaucoma and primary angle-closure glaucoma. Arch Ophthalmol 120: 1636–1643. 12470136 10.1001/archopht.120.12.1636

[19] Uchida H, Yamamoto T, Tomita G, Kitazawa Y (1999) Peripapillary atrophy in primary angle-closure glaucoma: a comparative study with primary open-angle glaucoma. Am J Ophthalmol 127: 121–128. 10030551 10.1016/s0002-9394(98)00318-3

[20] Lee PJ, Liu C, Wojciechowski R, Bailey-Wilson JE, Cheng CY (2010) Structure–Function Correlations Using Scanning Laser Polarimetry in Primary Angle-Closure Glaucoma and Primary Open-Angle Glaucoma. Am J Ophthalmol 149: 817–825. 10.1016/j.ajo.2009.12.007 20202618 PMC2866157

[21] Cai F, Gao L, Gong H, Jiang F, Pei C, Zhang X, et al (2015) Network centrality of resting-state fMRI in primary angle-closure glaucoma before and after surgery. PLoS One 10: e0141389 10.1371/journal.pone.0141389 26506229 PMC4624709

[22] Sato JR, Salum GA, Gadelha A, Vieira G, Zugman A, Picon FA, et al (2015) Decreased centrality of subcortical regions during the transition to adolescence: a functional connectivity study. Neuroimage 104: 44–51. 10.1016/j.neuroimage.2014.09.063 25290886

[23] Biswal B, Yetkin FZ, Haughton VM, Hyde JS (1995) Functional connectivity in the motor cortex of resting human brain using echo-planar MRI. Magn Reson Med 34: 537–541. 8524021 10.1002/mrm.1910340409

[24] Chao-Gan Y, Yu-Feng Z (2010) DPARSF: A MATLAB Toolbox for “Pipeline” Data Analysis of Resting-State fMRI. Front Syst Neurosci 4: 13 10.3389/fnsys.2010.00013 20577591 PMC2889691

[25] Murphy K, Birn RM, Handwerker DA, Jones TB, Bandettini PA (2009) The impact of global signal regression on resting state correlations: Are anti-correlated networks introduced? NeuroImage 44: 893–905. 10.1016/j.neuroimage.2008.09.036 18976716 PMC2750906

[26] Van Dijk KRA, Sabuncu MR, Buckner RL (2012) The influence of head motion on intrinsic functional connectivity MRI. Neuroimage 59: 431–438. 10.1016/j.neuroimage.2011.07.044 21810475 PMC3683830

[27] Yan CG, Wang XD, Zuo XN, Zang YF (2016) DPABI: Data Processing & Analysis for (Resting-State) Brain Imaging. Neuroinformatics 14: 339–351. 10.1007/s12021-016-9299-4 27075850

[28] Tillikainen L, Salli E, Korvenoja A, Aronen HJ (2006) A cluster mass permutation test with contextual enhancement for fMRI activation detection. Neuroimage 32: 654–664. 10.1016/j.neuroimage.2006.03.058 16769226

[29] Marchini J, Presanis A (2004) Comparing methods of analyzing fMRI statistical parametric maps. Neuroimage 22: 1203–1213. 10.1016/j.neuroimage.2004.03.030 15219592

[30] Ito Y, Shimazawa M, Chen YN, Tsuruma K, Yamashima T, Araie M, et al (2009) Morphological changes in the visual pathway induced by experimental glaucoma in Japanese monkeys. Exp Eye Res 89: 246–255. 10.1016/j.exer.2009.03.013 19341728

[31] Heng Hah M, Norliza Raja Omar R, Jalaluddin J, Fadzillah Abd Jalil N, Selvathurai A (2012) Outcome of trabeculectomy in hospital Melaka, Malaysia. Int J Ophthalmol 5: 384–388. 10.3980/j.issn.2222-3959.2012.03.26 22773993 PMC3388413

[32] Chadick JZ, Gazzaley A (2011) Differential coupling of visual cortex with default network or frontal-parietal network based on goals. Nat Neurosci 14: 830–832. 10.1038/nn.2823 21623362 PMC3125492

[33] Harmelech T, Friedman D, Malach R (2015) Differential Magnetic Resonance Neurofeedback Modulations across Extrinsic (Visual) and Intrinsic (Default-Mode) Nodes of the Human Cortex. J Neurosci 35: 2588–2595. 10.1523/JNEUROSCI.3098-14.2015 25673851 PMC6605617

[34] Jann K, Kottlow M, Dierks T, Boesch C, Koenig T (2010) Topographic Electrophysiological Signatures of fMRI Resting State Networks. PLoS One 5: e12945 10.1371/journal.pone.0012945 20877577 PMC2943931

[35] Wang K, Jiang T, Yu C, Tian L, Li J, Liu Y, et al (2008) Spontaneous activity associated with primary visual cortex: a resting-state FMRI study. CerebCortex 18: 697–704. 10.1093/cercor/bhm105 17602140

[36] Baumann O, Borra RJ, Bower JM, Cullen KE, Habas C, Ivry RB, et al (2015) Consensus Paper: The Role of the Cerebellum in Perceptual Processes. Cerebellum 14: 197–220. 10.1007/s12311-014-0627-7 25479821 PMC4346664

[37] Ding K, Liu Y, Yan X, Lin X, Jiang T (2013) Altered Functional Connectivity of the Primary Visual Cortex in Subjects with Amblyopia. Neural Plast 2013: 612086 10.1155/2013/612086 23844297 PMC3697400

