# Supplementary figures and images for: Intrinsic Functional Connectivity Alterations of the Primary Visual Cortex in Primary Angle-Closure Glaucoma Patients before and after Surgery: A Resting-State fMRI Study
Source: PLoS One. 2017 Jan 25;12(1):e0170598. doi: 10.1371/journal.pone.0170598 (PMC5266295; doi:10.1371/journal.pone.0170598)

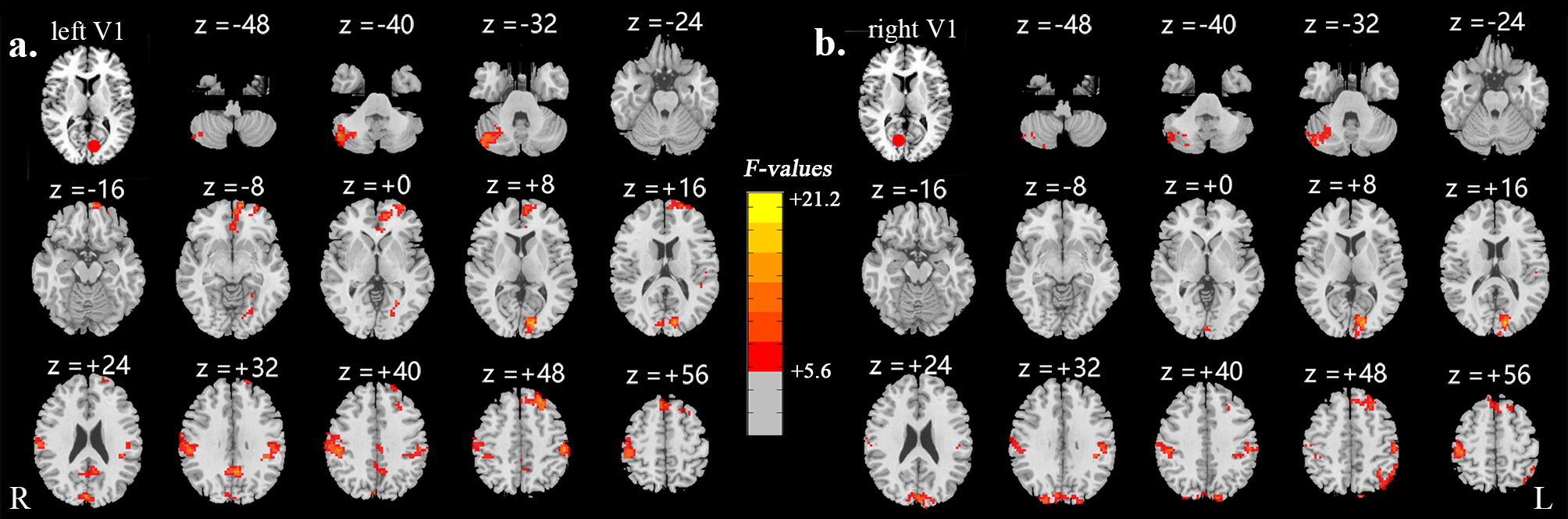

Supplement: S1 Fig — ANOVA of the iFC of the left V1 (a) and right V1 (b) in the HCs, and pre- and post-PACG patients (n = 9). The hot color indicates regions showing significant differences in iFC of the left (a) and right (b) V1 among the HCs, pre- and post-PACG patients (one-tailed voxel-level P < 0.01 and cluster-level P < 0.05 with GRF correction). These brain regions mainly included the right CPL, left SFG/MFG/ACC, left or right CUN/LIG/Ca/SOG, left PCU/PCC, and left or right POCG/IPL. Spatial distribution was visualized using DPABI slice viewer (http://rfmri.org/dpabi). (TIF) [file pone.0170598.s005.tif]

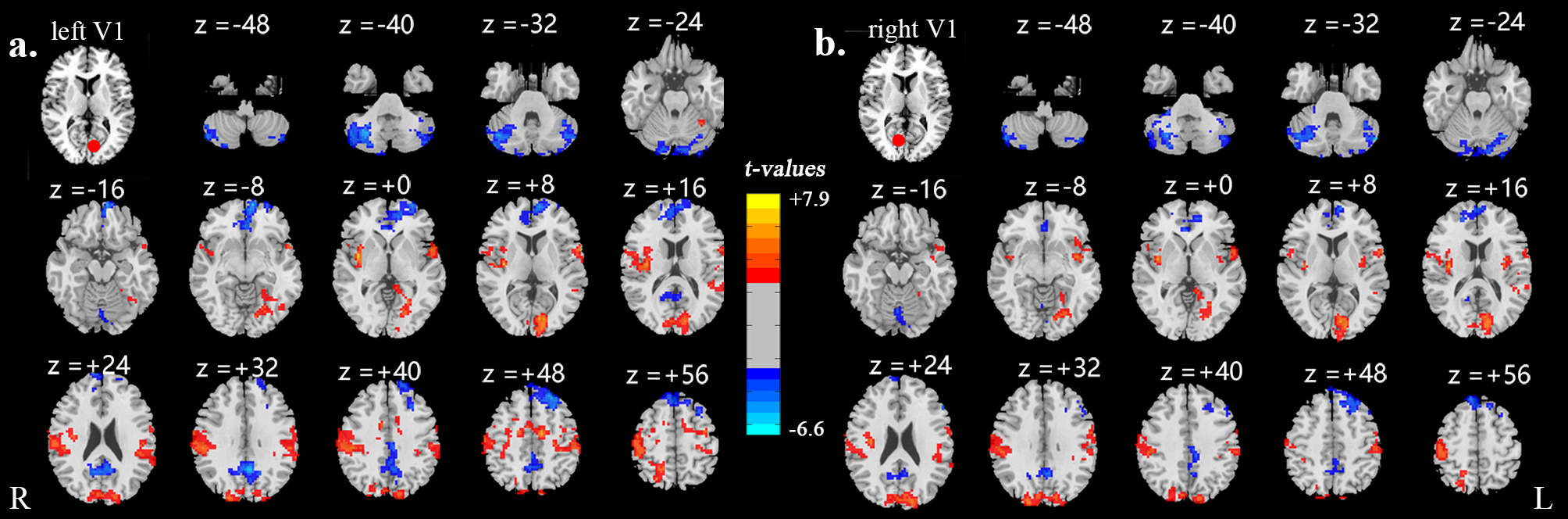

Supplement: S2 Fig — Alterations of iFC of the left V1 (a) and right V1 (b) between the HCs and post-PACG patients (n = 9). The hot (cool) color indicates increased (decreased) iFC of the left V1 (a) and right V1 (b) in the post-PACG patients compared with the HCs. Compared with the HCs, the post-PACG patients exhibited significantly increased iFC between the left or right CUN/right SOG/PCU/superior parietal lobule/left LIG/Ca, right INS/POCG/precentral gyrus (PRCG)/IPL, left POCG/PRCG/IPL/STG and the left V1; In addition, significantly decreased iFC was observed between the left or right CPL, left or right SFG/medial frontal gyrus/MFG/ACC, left or right PCU/PCC and the left V1. The iFC alterations of the right V1 was similar to those of the left V1. Spatial distribution was visualized using DPABI slice viewer (http://rfmri.org/dpabi). (TIF) [file pone.0170598.s006.tif]

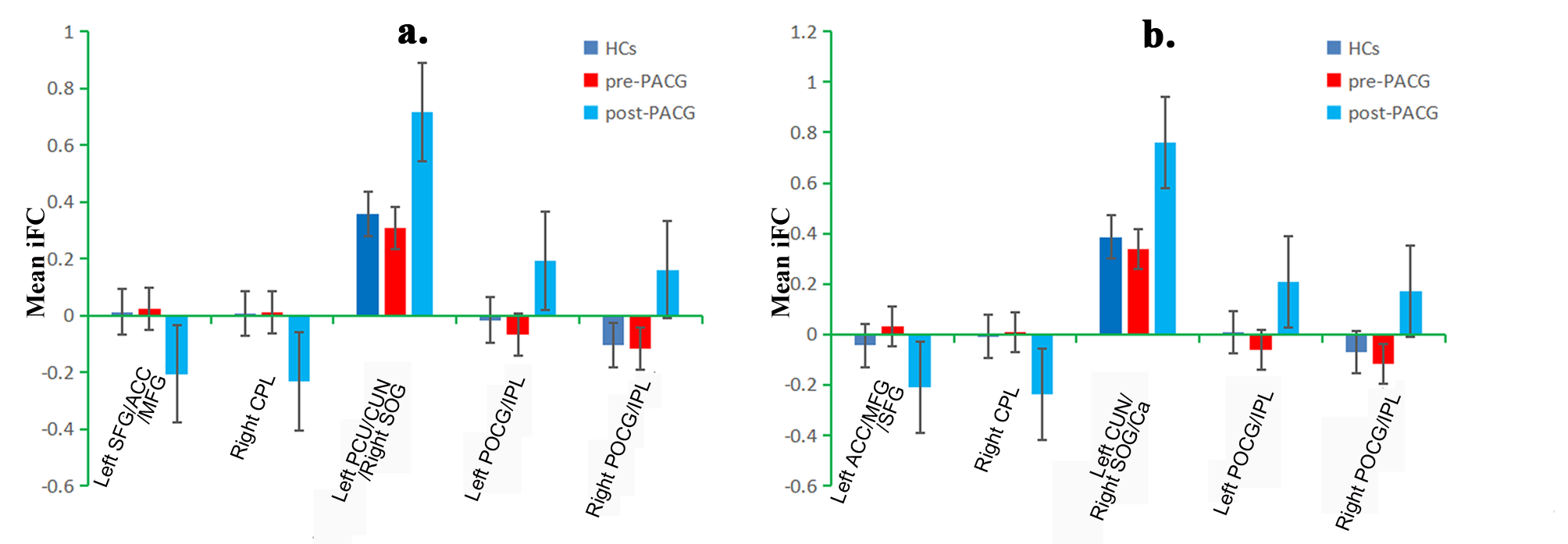

Supplement: S3 Fig — Bar plot of iFC of the left V1 (a) and right V1 (b) for the significant clusters in the post-PACG patients vs. pre-PACG patients vs. HCs (n = 9). (TIF) [file pone.0170598.s007.tif]
