# Supplementary material for: Intrinsic Functional Connectivity Alterations of the Primary Visual Cortex in Primary Angle-Closure Glaucoma Patients before and after Surgery: A Resting-State fMRI Study
Source: PLoS One. 2017 Jan 25;12(1):e0170598. doi: 10.1371/journal.pone.0170598 (PMC5266295; doi:10.1371/journal.pone.0170598)
Supplement: S1 Table — (DOC) [file pone.0170598.s008.doc]

**Table S1 Correlation analysis between the clincal indices and altered left V1-iFC coefficients in the** pre-PACG patients

|  | | RNFLT (μm) | A-C/D | V-C/D | IOP (mmHg) | VA | Disease duration (d) |
| --- | --- | --- | --- | --- | --- | --- | --- |
| R-CUN/Ca/LIG/PCC | Pearson correlation | .353 | -.163 | -.238 | -.032 | -.129 | .329 |
|  | Significant (two-tailed) | .083 | .438 | .251 | .881 | .540 | .108 |
|  | N | 25 | 25 | 25 | 25 | 25 | 25 |
| L-STG/MTG/IPL/HIP | Pearson correlation | -.083 | .027 | -.006 | -.029 | **.553^*^** | -.116 |
|  | Significant (two-tailed) | .693 | .898 | .976 | .889 | **.004** | .580 |
|  | N | 25 | 25 | 25 | 25 | **25** | 25 |
| L-IFG/EXN/PUT/INS | Pearson correlation | -.286 | .156 | .178 | -.248 | .275 | -.485 |
|  | Significant (two-tailed) | .165 | .458 | .394 | .232 | .183 | .014 |
|  | N | 25 | 25 | 25 | 25 | 25 | 25 |
| R-IPL | Pearson correlation | -.103 | -.018 | .083 | -.030 | **.643^*^** | -.317 |
|  | Significant (two-tailed) | .625 | .932 | .693 | .887 | **.001** | .123 |
|  | N | 25 | 25 | 25 | 25 | **25** | 25 |
| R-INS/PUT/EXN | Pearson correlation | -.178 | -.116 | -.040 | -.198 | .248 | -.330 |
|  | Significant (two-tailed) | .394 | .580 | .848 | .343 | .232 | .107 |
|  | N | 25 | 25 | 25 | 25 | 25 | 25 |

Note: *. significant correlation at 0. 0083 level with the Bonferroni correction (two-tailed).
