# Supplementary material for: Intrinsic Functional Connectivity Alterations of the Primary Visual Cortex in Primary Angle-Closure Glaucoma Patients before and after Surgery: A Resting-State fMRI Study
Source: PLoS One. 2017 Jan 25;12(1):e0170598. doi: 10.1371/journal.pone.0170598 (PMC5266295; doi:10.1371/journal.pone.0170598)
Supplement: S2 Table — (DOC) [file pone.0170598.s009.doc]

**Table S2 Correlation analysis between the clincal indices and altered right V1-iFC coefficients in the** pre-PACG patients

|  | | RNFLT (μm) | A-C/D | V-C/D | IOP (mmHg) | VA | Disease duration (d) |
| --- | --- | --- | --- | --- | --- | --- | --- |
| L-CAL/PCC/Ca/R-LIG | Pearson correlation | .230 | .057 | -.061 | .096 | -.287 | .360 |
|  | Significant (two-tailed) | .268 | .786 | .772 | .647 | .164 | .077 |
|  | N | 25 | 25 | 25 | 25 | 25 | 25 |
| L-STG/MTG/HIP | Pearson correlation | -.118 | .018 | .098 | .019 | .512 | -.013 |
|  | Significant (two-tailed) | .573 | .932 | .640 | .927 | .009 | .951 |
|  | N | 25 | 25 | 25 | 25 | 25 | 25 |
| L-EXN/PUT/INS/IFG | Pearson correlation | -.372 | .088 | .167 | -.167 | .352 | -.254 |
|  | Significant (two-tailed) | .067 | .677 | .424 | .425 | .084 | .220 |
|  | N | 25 | 25 | 25 | 25 | 25 | 25 |
| R-INS/PUT/EXN | Pearson correlation | -.172 | -.201 | -.112 | -.127 | .315 | -.224 |
|  | Significant (two-tailed) | .412 | .335 | .595 | .544 | .126 | .281 |
|  | N | 25 | 25 | 25 | 25 | 25 | 25 |

Note: *. significant correlation at 0. 0083 level with the Bonferroni correction (two-tailed).
