# Supplementary material for: Intrinsic Functional Connectivity Alterations of the Primary Visual Cortex in Primary Angle-Closure Glaucoma Patients before and after Surgery: A Resting-State fMRI Study
Source: PLoS One. 2017 Jan 25;12(1):e0170598. doi: 10.1371/journal.pone.0170598 (PMC5266295; doi:10.1371/journal.pone.0170598)
Supplement: S3 Table — (DOC) [file pone.0170598.s010.doc]

**Table S3 Correlation analysis between the IOP and altered V1-iFC coefficients in the post-PACG patients**

| **The altered right V1-iFC coefficient** | | **IOP** |
| --- | --- | --- |
| Left ACC/MFG/SFG | Pearson correlation | .216 |
|  | Significant (two-tailed) | .577 |
|  | N | 9 |
| Right CPL | Pearson correlation | .003 |
|  | Significant (two-tailed) | .994 |
|  | N | 9 |
| Left CUN/Right SOG/Ca | Pearson correlation | -.246 |
|  | Significant (two-tailed) | .523 |
|  | N | 9 |
| Left POCG/IPL | Pearson correlation | .004 |
|  | Significant (two-tailed) | .991 |
|  | N | 9 |
| Right POCG/IPL | Pearson correlation | .275 |
|  | Significant (two-tailed) | .474 |
|  | N | 9 |

Note: *. significant correlation at 0. 05 level (two-tailed).

| **The altered left V1-iFC coefficient** | | **IOP** |
| --- | --- | --- |
| Left SFG/ACC/MFG | Pearson correlation | -.015 |
|  | Significant (two-tailed) | .969 |
|  | N | 9 |
| Right CPL | Pearson correlation | -.100 |
|  | Significant (two-tailed) | .798 |
|  | N | 9 |
| Left PCU/CUN/Right SOG | Pearson correlation | -.229 |
|  | Significant (two-tailed) | .553 |
|  | N | 9 |
| Left POCG/IPL | Pearson correlation | .087 |
|  | Significant (two-tailed) | .823 |
|  | N | 9 |
| Right POCG/IPL | Pearson correlation | .322 |
|  | Significant (two-tailed) | .398 |
|  | N | 9 |
